# Cost effectiveness of outpatient lumbar discectomy

**DOI:** 10.1186/s12962-021-00272-w

**Published:** 2021-03-26

**Authors:** Daniela Linhares, João A. Fonseca, Manuel Ribeiro da Silva, Filipe Conceição, António Sousa, Bernardo Sousa-Pinto, Nuno Neves

**Affiliations:** 1 Orthopedics Department, Centro Hospitalar e Universitário de São João, Porto, Portugal; 2 MEDCIDS – Department of Community Medicine, Information and Health Decision Sciences, Faculty of Medicine, University of Porto, Porto, Portugal; 3 CINTESIS, Center for Research in Health Technology and Information Systems, Faculty of Medicine, University of Porto, Porto, Portugal; 4 CUF Porto Hospital, Porto, Portugal; 5 i3S - Instituto de Investigação e Inovação Em Saúde, University of Porto, Porto, Portugal; 6 INEB - Instituto Nacional de Engenharia Biomédica, University of Porto, Porto, Portugal; 7 Surgery Unit, Centro Hospitalar E Universitário de São João, Porto, Portugal; 8 Surgery and Physiology Department, Faculty of Medicine, University of Porto, Porto, Portugal

**Keywords:** Diskectomy, Intervertebral Disc Displacement, Outpatients, Patient Reported Outcome Measures, Cost–Benefit Analysis, Economics

## Abstract

**Background:**

Microdiscectomy is the most commonly performed spine surgery and the first transitioning for outpatient settings. However, this transition was never studied, in what comes to cost-utility assessment. Accordingly, this economic study aims to access the cost-effectiveness of outpatient lumbar microdiscectomy when compared with the inpatient procedure.

**Methods:**

This is a cost utility study, adopting the hospital perspective. Direct medical costs were retrieved from the assessment of 20 patients undergoing outpatient lumbar microdiscectomy and 20 undergoing inpatient lumbar microdiscectomy Quality-adjusted life-years were calculated from Oswestry Disability Index values (ODI). ODI was prospectively assessed in outpatients in pre and 3- and 6-month post-operative evaluations. Inpatient ODI data were estimated from a meta-analysis. A probabilistic sensitivity analysis was performed and incremental cost-effectiveness ratio (ICER) calculated.

**Results:**

Outpatient procedure was cost-saving in all models tested. At 3-month assessment ICER ranged from €135,753 to €345,755/QALY, higher than the predefined threshold of €60,000/QALY gained. At 6-month costs were lower and utilities were higher in outpatient, overpowering the inpatient procedure. Probabilistic sensitivity analysis showed that in 65% to 73% of simulations outpatient was the better option. The savings with outpatient were about 55% of inpatient values, with similar utility scores. No 30-day readmissions were recorded in either group.

**Conclusion:**

This is the first economic study on cost-effectiveness of outpatient lumbar microdiscectomy, showing a significant reduction in costs, with a similar clinical outcome, proving it cost-effective.

**Supplementary Information:**

The online version contains supplementary material available at 10.1186/s12962-021-00272-w.

## Introduction/Background

Lumbar disc herniation results from disc degeneration, with protrusion or extrusion of the nucleus pulposus. It can be asymptomatic or lead to a myriad of symptoms, forcing patients to seek medical treatment [1, 2]. While the conservative approach is the mainstay, upon its failure, surgery is associated with successful outcome [1]. Although many surgical procedures have been described for the treatment of herniated lumbar discs, lumbar discectomy is not only safe but also the simplest and most effective [3]. Due to its simplicity and low rate of complications, discectomy comprises 70–90% of all outpatient procedures performed in spinal surgery [4].

Many procedures have emerged from the traditional open discectomy, but microdiscectomy (MD) has been shown to provide a faster relief of pain, being nowadays the most common spinal surgery performed in United States, with more than 300,000 annual procedures [5]. As a result, MD represents a substantial burden to healthcare systems [6, 7] and policies for cost reduction—such as ambulatory surgeries—are needed.

In fact, the literature depicts descriptions of outpatient discectomies since the 1980s [8] and MD was the first major spine surgery to transition to the ambulatory setting, with some centers performing almost half of these procedures in an outpatient basis [9]. However, many countries and centers still experience barriers in ambulatory transition. Actually, as described by the World Health Organization (WHO), economic advantages for hospitals from inpatient procedures, lack of educational programs, and absence of adapted facilities and community support can delay the progression to day surgeries [10]. Adequate scientific background and reassurance is needed to support this transition and reduce misinformation [10].

So, and although general outpatient procedures appear to be safe and effective [11], in the specific case of lumbar MD, a wider adoption of outpatient procedures may be precluded by insufficient evidence on the effectiveness, safety and economic savings of ambulatory MD compared to inpatient MD. Therefore, this cost-utility study aims to compare inpatient with outpatient lumbar MD regarding both its costs and effectiveness in adult patients with lumbar disc herniation and sciatica.

## Methods

### Study design

This is a cost-utility study, corresponding to a full economic evaluation comparing both costs and effectivity in patients undergoing MD in the outpatient *versus* in the inpatient setting in a Portuguese National Healthcare System hospital. Effectivity is presented as quality-adjusted life-years (QALYs), with the number of QALYs calculated by the product between life years and utilities. We followed the hospital perspective, considering direct hospital costs.

Uncertainty was explored via an one-way deterministic sensitivity analysis and probability sensitivity analyses.

The study was approved by a hospital ethics committee in May 25th 2017.

### Costs

Costs were assessed from two cohorts of patients treated in the same spine center of a central Portuguese hospital. Accordingly, specific data from 20 outpatients and 20 inpatients undergoing MD with single excision of herniated intervertebral disk was gathered. To be included in either group, patients had to present clinical complaints compatible with lumbar disc herniation and with confirmation of clinical findings by radiological studies (computed tomography and/or magnetic resonance imaging). Patients were excluded if they presented: (1) comorbidities precluding outpatient surgery; (2) social conditions precluding outpatient surgery (i.e. living alone or far from the hospital, psychiatric conditions); (3) need for additional spine surgical procedures other than single excision of herniated intervertebral disk; or (4) previous lumbar spine surgery. Upon inclusion, all patients were submitted to a lumbar MD by the same surgical team.

Costs were defined as the sum of direct hospital costs related with inpatient and outpatient procedures. For outpatients, we quantified operatory room (OR) costs, including costs related with (1) staff and OR occupation; (2) used drugs; (3) supplies used in that particular intervention; and (3) other costs. In addition, we quantified costs related with eventual 30-days readmissions. Such costs were also quantified for inpatients, among whom costs related to hospital stay were also added. The latter include staff-, drug- and supplies- (i.e., bandages, disposable wearing, etc.) related costs. For both inpatients and outpatients, we retrieved other costs related with water supply, electricity, telephone services, administrative issues, etc. Both groups of patients had the first post-operative appointment 2 weeks after the surgery and follow a similar medical follow-up.

Regarding outpatients, we prospectively analyzed a consecutive sample of 20 patients, presenting to our spine center between 2017 and 2018, with clinical pain and disability due to radiologically-identified lumbar disc herniation that fulfilled the above-mentioned criteria. A pre-defined outpatient protocol was followed, with patients being submitted to a pre-operative anesthetic evaluation and provided with aseptic sponges to bath in the morning before the procedure. After surgery, all patients were discharged in the same day, less than 12 h after the procedure and received a pre-defined analgesic protocol. To assess complications in the immediate post-operative period, a physician performed a telephone call up to 24 h after discharge, with the patient being directed to an emergency appointment if any complication was suspected.

Assessed inpatients consisted of a sample of 20 individuals, fulfilling the above-mentioned criteria, with similar age and gender to those of outpatients, and who were retrospectively selected from patients submitted to MD in the same spine center.

Despite the literature describing a variable length of stay among MD patients in real life scenarios, there is an overall agreement among spine surgeons that an uncomplicated inpatient MD would only need a one-day admission [12]. As a result, we not only performed this economic evaluation study estimating inpatient costs as observed (irrespective of the admission time), but also performed a sensitivity analyses considering the scenario of all patients being only admitted for one day. To do so, costs for inpatients that stayed for longer periods were re-calculated for those expected in a one-day admission period.

### Utilities

### Utilities were estimated from the Oswestry Disability Index (ODI)

Outpatients were prospectively evaluated pre-operatively and three and six months post-operatively, with ODI being assessed in each evaluation, along with the overall visual analogue scale of pain (VAS), back pain VAS (BP-VAS), and leg pain VAS (LP-VAS).

Since inpatient data from our center were collected retrospectively, ODI data were retrieved from the literature. To do so, we performed a comprehensive search on MEDLINE from 2018 to 2020 (limited to humans and systematic reviews), using a combination of the search terms: “lumbar”, “hernia”, “protrusion”, “extrusion”, “discectomy” and “microdiscectomy”. We specifically searched for studies on lumbar MD, displaying ODI data on pre- and post-operative assessments at 3 and/or 6 months assessments after the surgical procedure. Of a total of 110 retrieved references, we identified one systematic review with meta-analysis fulfilling all eligibility criteria and utilities were estimated from its data on ODI [13].

QALYs were estimated based on three and 6-months utilities, adjusted for baseline values, using two different approaches—the area under the curve (AUC) and change from baseline (CfB) approach [14]. For outpatients, average and standard-deviation values for QALYs based on each approach were estimated using patient-level data. For inpatients, such values were estimated following Bayesian methods—a random-effects Bayesian meta-analysis was performed to obtain pooled baseline utilities and mean utilities differences, which were then used in the same Bayesian model to estimate the average and standard-deviation values for QALYs (via assessment of the posterior distributions) following the AUC and CfB approaches. Uninformative prior distributions were used in Bayesian models both for the effect size measures and for the tau parameters (dnorm (0,0.00001) and dunif (0,10), respectively).

### Data analysis

Categorical variables were described using absolute and relative frequencies, while continuous variables were described using means and standard-deviations. Categorical variables were compared using the chi-square test, while continuous variables were compared using the independent samples t-test and its non-parametric counterparts.

To assess for cost-effectiveness, we estimated incremental cost-effectiveness ratios (ICER), consisting of the difference between costs (i.e., outpatient minus inpatient costs) dividing by the difference in QALYs (i.e., outpatient minus inpatient QALYs). To account for uncertainty, we performed one-way deterministic sensitivity analysis, testing the effect of changing one variable at each time according to a prespecified range of values—observed minimum and maximum values were used for costs, while for QALYs (which were estimated by Bayesian values), the minimum and maximum values used for sensitivity analyses were obtained after 10,000 simulations based on their distributions. In addition, to explore uncertainty we conducted probabilistic sensitivity analysis via Monte Carlo simulation methods—we ran 10,000 simulations in which we allowed each input variable to vary according to a probability distribution. A treatment choice was regarded as cost-effective if its ICER was lower than the defined willingness to pay (WTP) per gained QALY. As indicated by WHO, The WTP was defined at 3 times the Portuguese per capita gross domestic product (GDP) [15]. Using the last available International Monetary Fund values (2019), this corresponds to a WTP value of €60,000 [16]. This probability sensitivity analysis was performed for both inpatient’s observed admission time and for one day only. Frequentist statistical analysis was performed using SPSS v26 (IBM SPSS Statistics, NY. Bayesian models were performed using rjags package for software R (version 4.0). Probabilistic sensitivity analysis was performed using TreeAgePro 2019 (TreeAge Software, Williamstown, MA).

## Results

### Costs data

Costs were retrieved from 20 outpatients and 20 inpatients submitted to lumbar MD, in whom no significant differences were found for any assessed sociodemographic characteristics (Table 1). Inpatients length of stay averaged 2.5 days. No 30-day readmission was recorded. No loss of follow-up was observed in the six-month assessment period for the outpatients (Table 1).

**Table 1:** Demographic data of inpatient and outpatient

	Setting		p value
	Inpatient (N = 20)	Outpatient (N = 20)	
Age at surgery	46.8 ± 11.0	44.9 ± 10.87	0.586
N of Females-N (%)	8 (40%)	10 (50%)	0.525
Level-N (%)			0.524
L2L3	1 (5%)		
L4L5	12 (60%)	11 (55%)	
L5S1	7 (35%)	9 (45%)	
Side-N (%)			0.626
Left	12 (60%)	12 (60%)	
Right	8 (40%)	8 (40%)	
Hospital stay (days)	2.5 ± 0.89	0	< 0.001
30-day readmissions-N (%)	0	0	–

OR costs are costs related with the procedure. Hotel costs are costs related with hospital stay. N, number. OR, Operatory Room. Values are presented as mean ± standard deviation

Overall hospital costs averaged €630.1 ± 18.4 per patient in outpatients and, €1477.7 ± 207.0 per patient in inpatients (*p* < 0.001). This represented an average save of €847.52 (95% confidence interval (CI) = €750.36–944.67), corresponding to a cost reduction of 55% (95% CI = 35.8%–66.9%). OR costs were also significantly higher in inpatients compared to outpatients (average €883.7 *versus* €630.1, p < 0.001) (Table 2).

**Table 2:** Costs associated with inpatient and outpatient interventions

	Setting		p value
	Inpatient (N = 20)	Outpatient (N = 20)	
Operatory Room Costs	€883.73	€630.14	< 0.001
Drugs and related	€44.39	€24.63	< 0.001
OR supplies	€79.54	€69.43	0.014
Staff	€560.08	€468.38	< 0.001
Other costs	€199.72	€67.70	< 0.001
Hotel Costs	€593.93	−	
Drugs and related	€39.73	−	
Medical supplies	€57.28	−	
Staff	€355.53	−	
Other costs	€112.57	−	
Diagnostic tests	€28.83	−	
Overall Costs	€1477.66	€630.14	< 0.001

OR costs are costs related with the procedure. Hotel costs are costs related with hospital stay. N, number. OR, Operatory Room. Values are presented as mean

Considering an inpatient admission time of one day the overall costs would be of €1128.2 ± 25.5, with a mean hotel cost of €244.5 ± 1.9.

### Utility data

Data on outpatient pre-operative, 3-month and 6-month assessments are available at Additional file 1. Between sequential assessments, a significant improvement was obtained in all outcomes (all p < 0.001), including ODI changes. All ODI and VAS inpatient changes were significantly higher than minimal clinical important differences. Inpatient ODI were retrieved from a systematic review and meta-analysis [17], with 6 primary studies displaying data at 3-month ODI, and 4 studies at 6-month ODI. Data on inpatient and outpatient utility values for the 3 and 6-month assessments are available at Table 3.

**Table 3:** Utilities estimated from inpatient and outpatient assessments

Utilities	Baseline	3 months	6 months
Outpatient	0.508 ± 0.098	0.646 ± 0.092	0.720 ± 0.063
Inpatient at 3-month	0.492 ± 0.065	0.678 ± 0.088	–
Inpatient at 6-month	0.474 ± 0.107	−	0.695 ± 0.143

Values for inpatient are a result of meta-analysis including 6 studies at 3-months and 4 studies at 6-months. Values are presented as mean ± standard deviation

At the 3-month assessment, MD is associated with a gain of 0.14 or 0.02 QALY in the outpatient setting and 0.15 or 0.02 in the inpatient setting, respectively depending on whether the AUC or the CfB approach is being considered. At 6-months, these gains were of 0.32 (AUC) or 0.06 (CfB) QALY for inpatients, and 0.29 (AUC) or 0.06 QALY (CfB) for outpatients (Table 4).

**Table 4:** Input variables included in economic evaluation model

Input variable	Mean ± SD	Type of distribution	Information source
Costs–Euro– ± 2.5 days admission			Primary (our sample)
Surgical costs in ambulatory setting	630.1 ± 18.4	Gamma	
Surgical costs in hospitalization setting	883.7 ± 26.9	Gamma	
Hotel costs in hospitalization setting	593.5 ± 211.1	Gamma	
Costs–Euro–1 day admission			Primary (our sample)
Surgical costs in ambulatory setting	630.1 ± 18.4	Gamma	
Surgical costs in hospitalization setting	883.7 ± 26.9	Gamma	
Hotel costs in hospitalization setting	244.5 ± 1.9	Gamma	
QALYs in ambulatory setting			Primary (our sample)
Based on 3 months ODI data [AUC method]	0.144 ± 0.022	Gamma	
Based on 3 months ODI data [CfB method]	0.017 ± 0.010	Gamma	
Based on 6 months ODI data [AUC method]	0.315 ± 0.040	Gamma	
Based on 6 months ODI data [CfB method]	0.061 ± 0.026	Gamma	
QALYs in hospitalization setting			Meta-analysis
Based on 3 months ODI data [AUC method]	0.146 ± 0.018	Gamma	
Based on 3 months ODI data [CfB method]	0.023 ± 0.007	Gamma	
Based on 6 months ODI data [AUC method]	0.293 ± 0.067	Gamma	
Based on 6 months ODI data [CfB method]	0.055 ± 0.021	Gamma	

OR costs are costs related with the procedure. Hotel costs are costs related with hospital stay. SD, standard deviations. OR, operatory Room; AUC, Area under the curve; CfB, Change from Baseline; ODI, Oswestry Disability Index; QALY, Quality-adjusted life year

### Cost-utility analysis

Considering data from 3-month post-operative assessments, inpatient MD was associated both with higher costs and small QALY gains, resulting in an ICER of €345,755.1/QALY gained (AUC approach) or €135,753.2/QALY gained (CfB approach) (Table 5). In both cases, ICER are higher than the WTP threshold of €60,000/QALY gained, rendering inpatient surgery not cost-effective.

**Table 5:** Cost-utility analysis for observed admission time and 1-day admission time

	3-month		6-month	
	AUC	CfB	AUC	CfB
Observed admission time				
ICER (outpatient vs inpatient)	€345,755.1/QALY	€135,753.2/QALY	–	–
%Simulations outpatient better than inpatient	65.2%	73.0%	68.9%	71.8%
1-day admission time				
ICER (outpatient vs inpatient)	€207,541.7/QALY	€80,338.7/QALY	–	–
% simulations outpatient better than inpatient	58.4%	54.4%	66.3%	66.4%

ICER values for 6-month assessment are not presented because, since outpatient is associated both with lower costs and higher utility gains, inpatient was dominated. AUC, Area under the curve; CfB, Change from Baseline; ICER, Incremental Cost-Effectiveness Ratio; QALY, Quality-adjusted life year

**Fig. 1:**
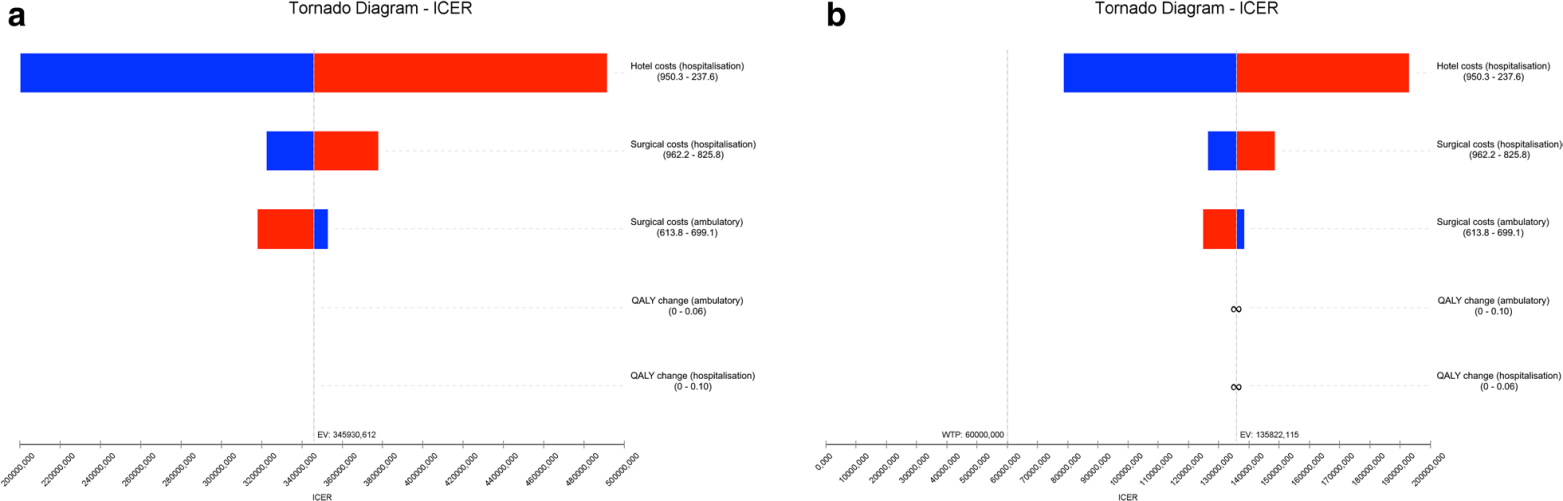
Incremental cost-effectiveness ratio (ICER) tornado diagram for one-way sensitivity analyses at 3-month assessment with inpatient costs calculated for the observed admission time. The minimum and maximum values for each input variable are presented in brackets and the dashed line represents the willingness-to-pay threshold. **a** Sensitivity analyses with QALYs change computed based on the area under curve approach; **b** Sensitivity analyses with QALYs change computed based on the change from baseline approach

**Fig. 2:**
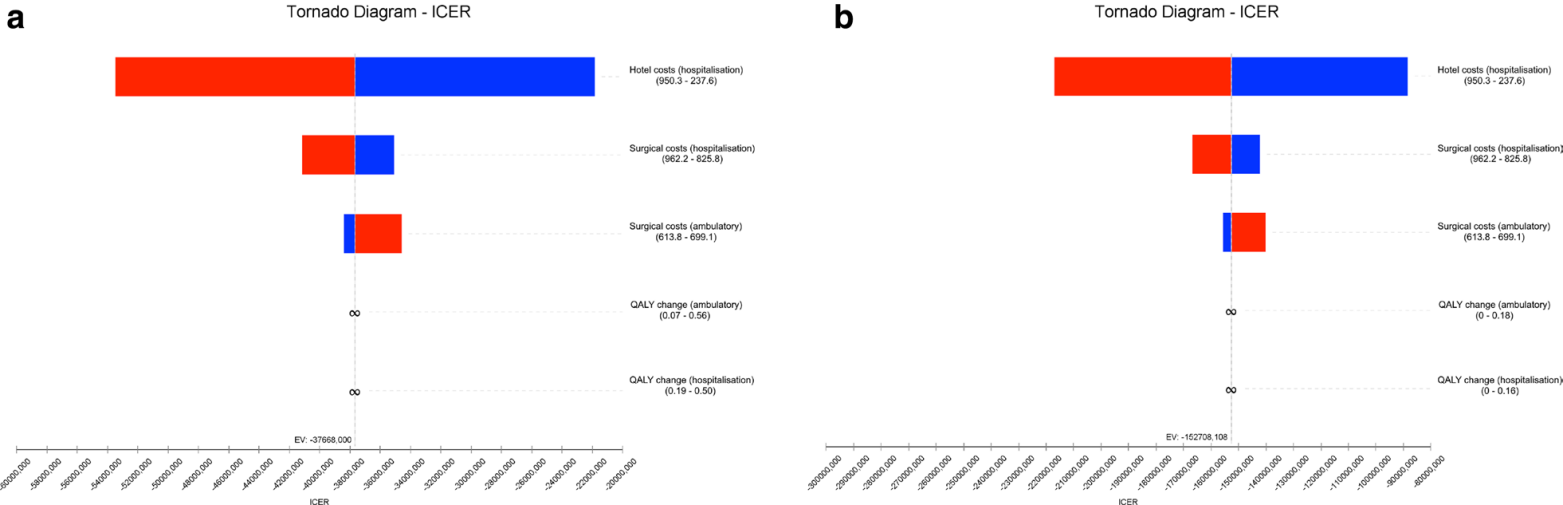
Incremental cost-effectiveness ratio (ICER) tornado diagram for one-way sensitivity analyses at 6-month assessment with inpatient costs calculated for the observed admission time. The minimum and maximum values for each input variable are presented in brackets and the dashed line represents the willingness-to-pay threshold. **a** Sensitivity analyses with QALYs change computed based on the area under curve approach; **b** Sensitivity analyses with QALYs change computed based on the change from baseline approach

One-way deterministic sensitivity analyses at 3-month assessments always resulted in ICER higher than the WTP threshold, indicating that the small utility gains in inpatient setting were not compensated by the underlying higher costs (Fig. 1). At 6-month assessments, the inpatient setting was always found to be the dominated strategy—it was always found to be associated with lower utilities and higher costs when compared to the outpatient setting, translating in negative ICER values (Fig. 2).

**Fig. 3:**
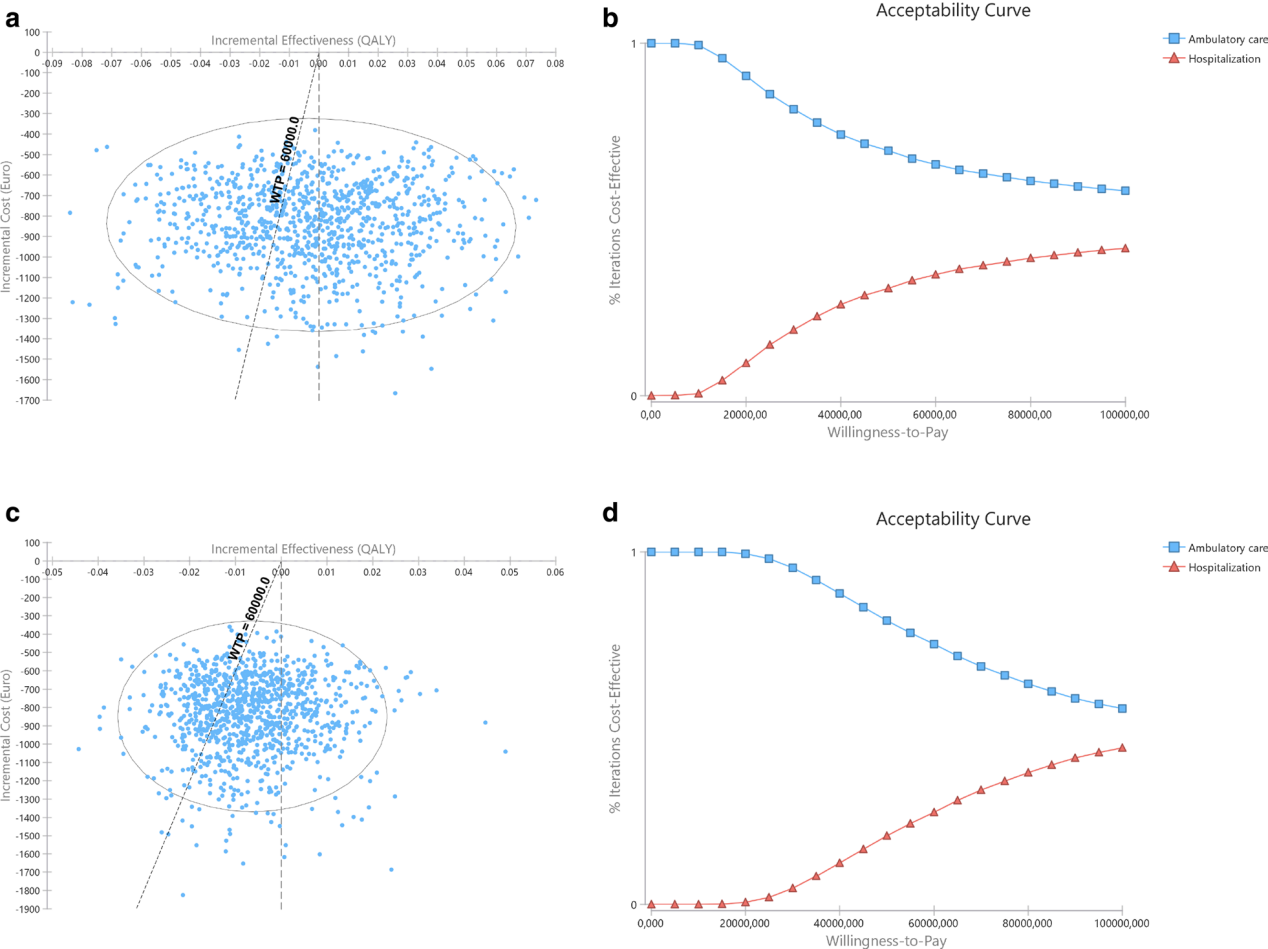
Results of probabilistic sensitivity analysis at 3-month assessment with inpatient costs calculated based in the observed admission time. **a** and **b** with QALYs computed based in area under curve; **c** and **d** based in change from baseline. Right (**a** and **c**): Incremental cost-effectiveness ratio scatterplots and 95% confidence interval ellipse. Each point represents a simulation, with indication of the mean incremental cost and effectiveness of outpatient compared to inpatient MD; the oblique dashed line represents the willingness-to-pay (WTP) threshold; Simulations represented to the left of the oblique dashed line (WTP line) represent those in which outpatient surgery was found to be less costly and less effective than inpatient surgery, with inpatient being the treatment of choice; Simulations to the right of the oblique dashed line (WTP line) and of the vertical line represent those in which outpatient surgery was found to be less costly and more effective than inpatient surgery with outpatient surgery being the treatment of choice. Between dashed lines are those in which outpatient was found to be less costly and less effective, but the effectiveness losses do not compensate the cost savings, and outpatient is the treatment of choice. In this model, and according to €60,000 WTP outpatient is better than inpatient in 65.2% (AUC) or 73.0% (CfB) of simulations. Left (**b** and **d**): Cost-effectiveness acceptability curve of outpatient versus inpatient. The Y-axis represents the probability of each comparator being cost-effective at a given willingness-to-pay (WTP) threshold, and ranges between 0 and 100%. Outpatient MD has been identified has cost effective throughout all different WTP thresholds depicted

In probabilistic sensitivity analysis, ambulatory MD was found to be the best strategy in 65.2% (AUC approach) and 73.0% (CfB approach) simulations (Fig. 3a and c, respectively, and Table 5).

**Fig. 4:**
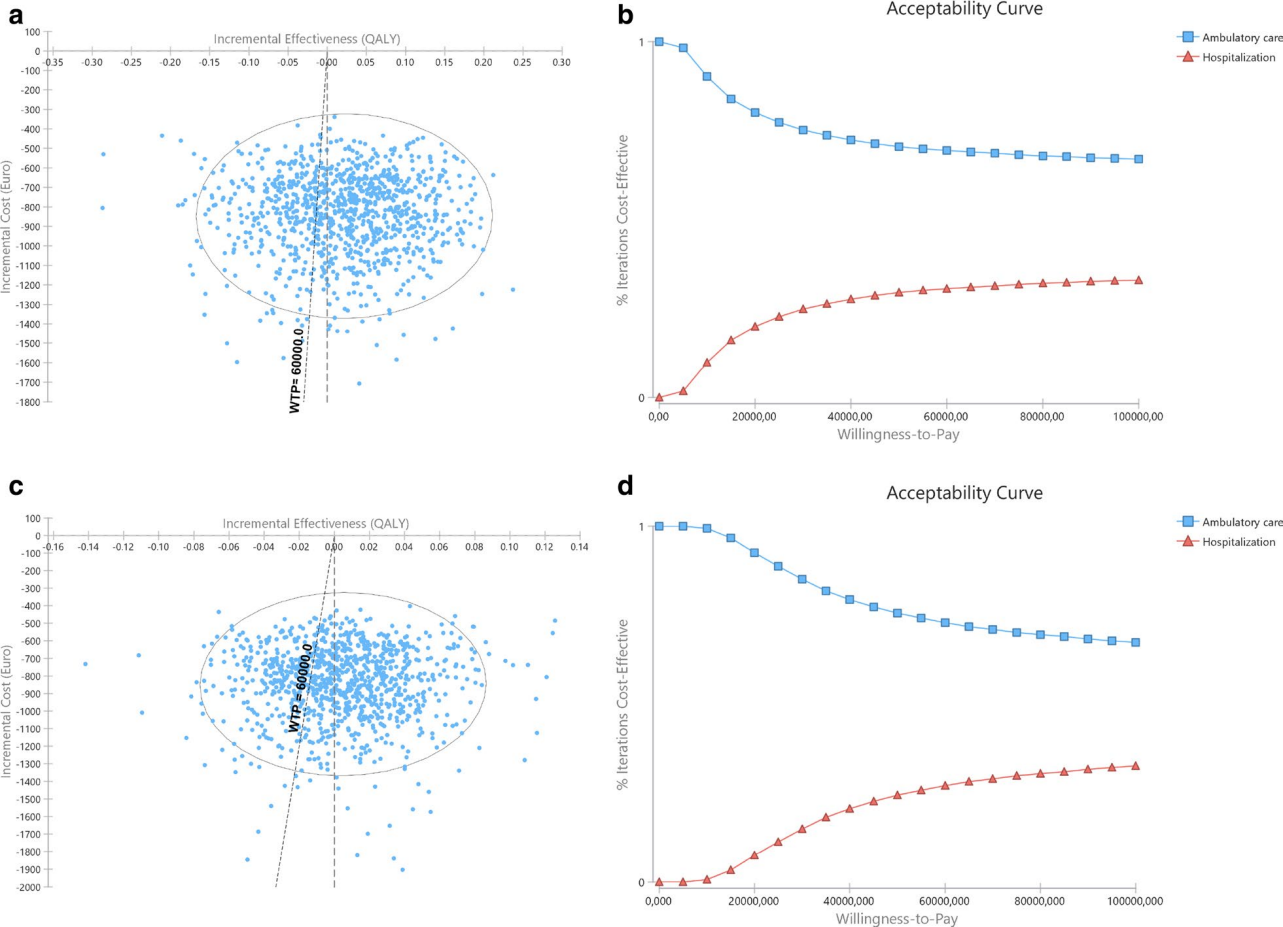
Results of probabilistic sensitivity analysis at 6-month assessment with inpatient costs calculated based in the observed admission time. **a** and **b** with QALYs computed based in area under curve; c and d based in change from baseline. Right (**a** and **c**): Incremental cost-effectiveness ratio scatterplots and 95% confidence interval ellipse. Each point represents a simulation, with indication of the mean incremental cost and effectiveness of outpatient compared to inpatient MD; the oblique dashed line represents the willingness-to-pay (WTP) threshold; Simulations represented to the left of the oblique dashed line (WTP line) represent those in which outpatient surgery was found to be less costly and less effective than inpatient surgery, with inpatient being the treatment of choice; Simulations to the right of the oblique dashed line (WTP line) and of the vertical line represent those in which outpatient surgery was found to be less costly and more effective than inpatient surgery with outpatient surgery being the treatment of choice. Between dashed lines are those in which outpatient was found to be less costly and less effective, but the effectiveness losses do not compensate the cost savings, and outpatient is the treatment of choice. In this model, and according to €60,000 WTP outpatient is better than inpatient in 68.9% (AUC) or 71.8% (CfB) of simulations. Left (**b** and **d**): Cost-effectiveness acceptability curve of outpatient versus inpatient. The Y-axis represents the probability of each comparator being cost-effective at a given willingness-to-pay (WTP) threshold, and ranges between 0 and 100%. Outpatient MD has been identified has cost effective throughout all different WTP thresholds depicted

At 6-month assessments, outpatient MD, associated both with lower costs and higher QALY gains—no ICER was, thus, calculated, since inpatient MD was dominated (Table 5). In probabilistic sensitivity analysis, ambulatory MD was found to be the best strategy in 68.9% (AUC) and 71.8% (CfB) simulations (Fig. 4a and c, respectively, and Table 5). Outpatient procedures remain cost-effective at 3 and 6-months, at any WTP between 0 and €100,000/QALY gained (Fig. 3b and d and Fig. 4b and d, respectively).

**Fig. 5:**
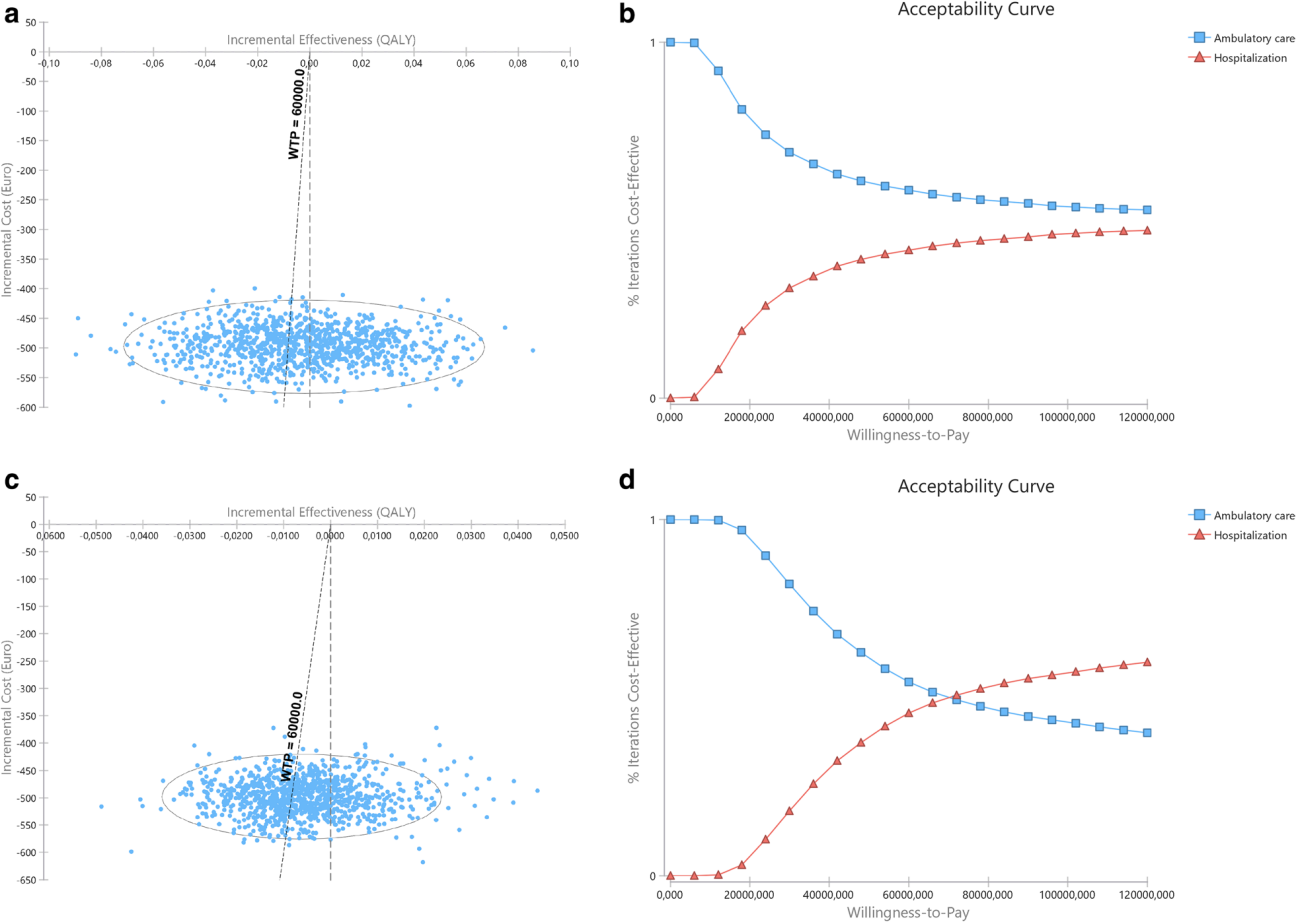
Results of probabilistic sensitivity analysis at 3-month assessment with inpatient costs calculated for one day of admission time. **a** and **b** with QALYs computed based in area under curve; c and d based in change from baseline. Right (**a** and **c**): Incremental cost-effectiveness ratio scatterplots and 95% confidence interval ellipse. Each point represents a simulation, with indication of the mean incremental cost and effectiveness of outpatient compared to inpatient MD; the oblique dashed line represents the willingness-to-pay (WTP) threshold; Simulations represented to the left of the oblique dashed line (WTP line) represent those in which outpatient surgery was found to be less costly and less effective than inpatient surgery, with inpatient being the treatment of choice; Simulations to the right of the oblique dashed line (WTP line) and of the vertical line represent those in which outpatient surgery was found to be less costly and more effective than inpatient surgery with outpatient surgery being the treatment of choice. Between dashed lines are those in which outpatient was found to be less costly and less effective, but the effectiveness losses do not compensate the cost savings, and outpatient is the treatment of choice. In this model, and according to €60,000 WTP outpatient is better than inpatient in 58.4% (AUC) or 54.4% (CfB) of simulations. Left (**b** and **d**): Cost-effectiveness acceptability curve of outpatient versus inpatient. The Y-axis represents the probability of each comparator being cost-effective at a given willingness-to-pay (WTP) threshold, and ranges between 0 and 100%. Outpatient MD has been identified has cost effective at the €60,000 WTP threshold

**Fig. 6:**
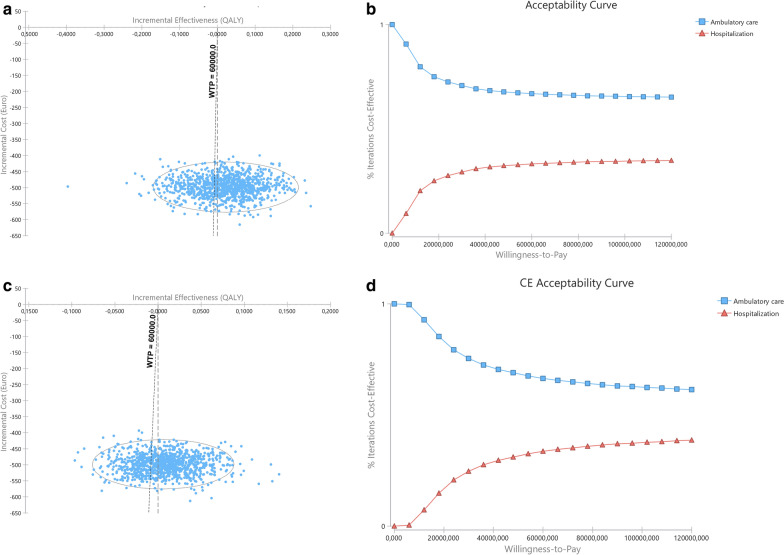
Results of probabilistic sensitivity analysis at 6-month assessment with inpatient costs calculated for one day of admission time. **a** and **b** with QALYs computed based in area under curve; **c** and **d** based in change from baseline. Right (**a** and **c**): Incremental cost-effectiveness ratio scatterplots and 95% confidence interval ellipse. Each point represents a simulation, with indication of the mean incremental cost and effectiveness of outpatient compared to inpatient MD; the oblique dashed line represents the willingness-to-pay (WTP) threshold; Simulations represented to the left of the oblique dashed line (WTP line) represent those in which outpatient surgery was found to be less costly and less effective than inpatient surgery, with inpatient being the treatment of choice; Simulations to the right of the oblique dashed line (WTP line) and of the vertical line represent those in which outpatient surgery was found to be less costly and more effective than inpatient surgery with outpatient surgery being the treatment of choice. Between dashed lines are those in which outpatient was found to be less costly and less effective, but the effectiveness losses do not compensate the cost savings, and outpatient is the treatment of choice. In this model, and according to €60,000 WTP outpatient is better than inpatient in 66.3% (AUC) or 66.4% (CfB) of simulations. Left (**b** and **d**): Cost-effectiveness acceptability curve of outpatient versus inpatient. The Y-axis represents the probability of each comparator being cost-effective at a given willingness-to-pay (WTP) threshold, and ranges between 0 and 100%. Outpatient MD has been identified has cost effective throughout all different WTP thresholds depicted

When inpatient costs for one admission day are considered, instead of costs for the observed admission period, 3-month ICER is of €207,541.7/QALY gained (AUC approach) or €80,338.7/QALY gained (CfB approach), remaining cost-effective at the defined WTP threshold of €60,000/QALY gained. Outpatient MD is the best strategy in 58.4% (AUC approach) and 54.4% (CfB approach) simulations (Fig. 5 and Table 5). At 6-month assessment, inpatient MD is still dominated, with MD being the best strategy in 66.3% (AUC) and 66.4% (CfB) simulations (Fig. 6 and Table 5).

## Discussion

Low back pain and related affections carry a cost of more than 100 billion dollars each year, only in United States, with disc disorders playing a substantial role in this amount [18]. To the best of our knowledge, our study was the first to show that outpatient lumbar MD is cost-effective. We observed that ambulatory MD was associated with a significant reduction of costs, with no relevant utility loss, resulting in ICER expressively higher than the defined WTP threshold. These results were consistently found in all analysis performed, including with different QALY estimation methods, considered time periods and WTP.

At 3-month evaluation, QALYs gained were slightly higher in inpatient setting, but such gains in effectiveness were not sufficient to compensate for the additional costs, with ICER of €135,753–345,755/QALY gained. However, at the 6-month assessment, even QALYs gained were observed to be higher in the outpatient setting, with the inpatient setting being a dominated strategy. These differences in QALYs gained, however, might not be relevant, and may rather result from an expected variation due to sample variability and to the disparity of sources chosen for clinical data analysis. In fact, similar to previous studies, we showed significant gains in VAS and ODI in the 3- and 6- month assessments following lumbar discectomy [5, 12], and those gains are not expected to differ between inpatient or outpatient procedures.

Although no previous study displayed results on MD transition to the outpatient setting, there are some examples on other surgical procedures, such as knee arthroplasty [19]. For the latter procedure, although outpatient surgery was proven cost-effective for the defined WTP, the inpatient procedure was found to be more effective [19]. On the contrary, our study showed a similar effectiveness for outpatient and inpatient lumbar MD, with a significant cost reduction associated with the former. This cost reduction agrees with the results of a former review that estimated average cost savings of 17.6% to 57.6% for outpatient orthopedic procedures when compared to similar procedures in hospitalized patients [20]. In fact, all analyzed costs were lower in outpatient setting, probably related with higher productivity rates and lower wasteful spending, leading to the decrease of individual costs depicted in Table 2.

This study has some limitations. We only considered direct medical costs, so that costs related with transportation, patient time, productivity and family assistance were not accounted. However, we expect no relevant differences in those indirect costs between compared groups, since all patients are walking and able to perform daily activities at discharge and acute complications are rare, what is reinforced by an absence of 30-day readmissions. Also, there is a limitation related with the observed admission time. Although it is expected that an inpatient submitted to an uncomplicated MD will only need one day of admission, many factors not directly related with the clinical condition and care may influence the length of stay [21]. To overcome these interferences, we computed the overall analysis for a hypothetic admission of one day, with outpatient surgery remaining cost-effective at both 3 and 6-month assessments. Another limitation is related with the fact that clinical data for inpatients were gathered from literature, based on a recent systematic review and meta-analysis [12]. Although we expect a small deviation from what would have happened in our sample, utilities were calculated from aggregated data from multiple studies that used similar samples and techniques. Nevertheless, the simulations performed under the probabilistic sensitivity analysis accounted for parameter uncertainty, as they considered the variability in the different variables included in the model, including the heterogeneity of inpatient utilities (Table 4). Therefore, it would not be expected that variations of this variable would be so drastic in order to draw our results to favoring inpatient surgery.

Another limitation concerns the criteria used in assortment of patients for outpatient treatment—overall, patients indicated for outpatient treatment tend to be younger and healthier, an already recognized selection bias in studies with patients undergoing outpatient spine surgery [11]. To account for this issue, in the present study, we selected a set of matched patients submitted to inpatient treatment following the same criteria applied for outpatient eligibility (and making sure they were operated by the same surgeon). Nevertheless, one should bear in mind that day surgery is reserved for a group of selected patients, with no significant comorbidities [20], and that some patients will continue to require lumbar MD in inpatient settings. Further limitations include the absence of data for a period longer than 6 months, and the fact that costs data were retrieved from a single country. Although the absolute costs are expected to be different in other settings, a similar magnitude of savings is predictable, as already demonstrated in the literature [20].

## Conclusion

In conclusion, we showed outpatient lumbar microdiscectomy to be cost-effective at the 3- and 6-months post-surgery, with a reduction of more than 50% costs when compared to the hospitalization setting, and similar utility gains. This is the first evidence of this clear benefit and should inform future health policies and clinical practice, advising for a global transition to ambulatory MD in patients eligible for this treatment modality.

## Supplementary Information

**Additional file 1:** Outpatient Clinical Assessments. Table with outpatient clinical assessments data.

## Data Availability

The datasets generated and/or analyzed during the current study are available from the corresponding author on reasonable request.
