# Supplementary material for: Cost effectiveness of outpatient lumbar discectomy
Source: Cost Eff Resour Alloc. 2021 Mar 26;19:19. doi: 10.1186/s12962-021-00272-w (PMC8004396; doi:10.1186/s12962-021-00272-w)
Supplement: Supplementary file 1 — Additional file 1: Outpatient Clinical Assessments. Table with outpatient clinical assessments data. [file 12962_2021_272_MOESM1_ESM.docx]

**Additional file 1**

|  | **Assessments** | | | **p-value** | | **p-value (variation vs MCID)** | |
| --- | --- | --- | --- | --- | --- | --- | --- |
|  | **Pre-operative** (N=20) | **3-mth** (N=20) | **6-mth** (N=20) | **Pre to 3-mth** | **3 to 6-mth** | **Pre-3 mth** | **3-6 mth** |
| **ODI (%)** | 53.1±18.87 | 26.4±17.84 | 12.2±12.16 | <0.001 | <0.001 | 0.002 | <0.001 |
| **Overall VAS** | 7.75±2.22 | 3.40±2.06 | 1.35±1.14 | <0.001 | <0.001 | <0.001 | <0.001 |
| **LP VAS** | 7.95±1.64 | 3.05±1.76 | 1.15±1.10 | <0.001 | <0.001 | <0.001 | <0.001 |
| **BP VAS** | 7.30±2.92 | 3.30±2.13 | 1.35±1.23 | <0.001 | <0.001 | <0.001 | <0.001 |

Outpatient Clinical Assessments. An improvement of leg pain (LP) VAS of 2 in 10 and a 15-point improvement in ODI were considered our minimal clinical important differences (MCID), as recommended by Federal Drug Administration.

mth: Months; N: number; ODI: Oswestry Disability Index; VAS: Visual Analog Scale; LP: Leg Pain; BP: Back Pain. Values are presented as mean±standard deviations.
